# Perioperative infarction during coronary bypass surgery: an attempt to refine the diagnostic criteria using data from a retrospective case–control study

**DOI:** 10.1177/03000605241306866

**Published:** 2025-01-27

**Authors:** Lars Niclauss, Liam-Kani Roulet, Piergiorgio Tozzi, Filip Dulguerov, Ziyad Gunga, Anna Nowacka, Matthias Kirsch

**Affiliations:** 1 Cardiovascular Surgery, University Hospital of Lausanne, Lausanne, Switzerland; 2 Faculty of Medicine, University of Lausanne, Lausanne, Switzerland

**Keywords:** Adverse outcome, perioperative myocardial infarction, coronary artery bypass graft surgery, type 5 myocardial infarction, postoperative high-sensitivity cardiac troponin T threshold, risk factor

## Abstract

**Objective:**

The definition of coronary artery bypass graft (CABG)-associated myocardial infarction (MI) is controversial because the postoperative increases in cardiac enzyme activities are multifactorial in origin.

**Methods:**

We performed a retrospective case–control study of patients who experienced perioperative MI (cardiac enzyme release, electrocardiographic changes, dysfunction on echocardiography) and those without ischemia to identify risk factors and enzyme activity thresholds.

**Results:**

The estimated incidence of CABG-associated MI was 2.8%. The risk factors were a family history of cardiovascular disease (odds ratio (OR) 2.8), tobacco abuse (OR 3.8), recent MI (OR 3.6), and triple-vessel disease (OR 2.8). The MI group showed higher mortality (OR 2.3), prolonged intubation (OR 3.1), and a prolonged stay in intensive care (OR 4.3). The type 5 MI threshold (10 times the upper limit of the reference range (URL)) was exceeded in 88.4% (troponin I) and 96% (high-sensitivity troponin T; hs-cTnT) of patients without ischemia.

**Conclusions:**

The frequent exceeding of conventional MI-indicating thresholds in patients without ischemia indicates their low specificity. An enzyme activity increase alone is of limited diagnostic value for perioperative MI, which is associated with greater mortality. Finally, the use of a higher threshold for hs-cTnT (>45 × URL) may increase its specificity for graft failure.

## Introduction

Cardiology and cardiac surgery societies are striving to clearly define perioperative coronary artery bypass grafting (CABG)-associated myocardial infarction (MI). The postoperative increases in the activities of cardiac-derived enzymes, which were included in the definition of type 5 MI in the 2018 European Society of Cardiology (ESC) guidelines, are influenced by several parameters, making it difficult to define a threshold that clearly indicates the presence of ischemic complications.^1^

Substantial cardiac enzyme release may also be the result of direct intraoperative mechanical or traumatic stress on cardiomyocytes, caused, for example, by heart squeezing or the dissection of target vessels, as well as generalized coronary hypoperfusion during induced cardiac arrest, which complicates the interpretation of a high troponin concentration as a marker of ischemia.^2^ In addition, changes on the electrocardiogram (ECG) and abnormal contractility on echocardiography, which are also included in the definition of type 5 MI, are limited in their specificity.^3^

The aim of the present study was to analyze the characteristics of patients with clearly documented permanent ischemic events that developed during CABG surgery, and compare these with a reference group of patients without ischemic complications, to identify risk factors and assess the potential effects of ischemic events on mortality and morbidity. In addition, we aimed to identify useful threshold values for circulating cardiac enzyme activities after surgery that are indicative of ischemia.

## Methods

### Ethics

The institutional review board (Swissethics, the Swiss Association of Research Ethics Committees: “CER, VD Commission cantonale d'éthique de la recherche sur l'être humain”) reviewed the study in accordance with international recommendations and approved it (protocol number 2020-02537; 1 April 2022). Data collection and processing were conducted in accordance with the World Medical Association’s Declaration of Helsinki − Ethical Principles for Medical Research Involving Human Subjects – of 1975, as revised in 2013. All the patient data were de-identified. All the participants either provided their written informed consent or were included in the study after an appropriate reflection period if they did not object, according to Article 34 of the Swiss Federal Act on Research involving human beings.

### Study design

We performed a retrospective case–control study of consecutive patients who underwent elective isolated on-pump CABG surgery at the authors’ institution between 2000 and 2020. The patients were assigned to either a target group that comprised those who had experienced a documented perioperative MI or a control group that comprised those who had not experienced corresponding pathologic perioperative ischemic events. The study is reported in accordance with the STROBE guidelines.^4^

### Inclusion/exclusion criteria

The inclusion criteria were admission for elective CABG surgery alone, the performance of an “on-pump” cardioplegic procedure, the completion of a 30-day follow-up period during which the patients remained in hospital or were examined as outpatients, and postoperative echocardiography performed no later than the fifth postoperative day.

Patients with postoperative reversible changes on their ECG were assigned to the perioperative MI group only if they had a second echocardiographic examination performed no earlier than the fifth postoperative day that showed persistent abnormalities in ventricular contraction.

Patients who underwent emergency or salvage interventions (as defined in the European System for Cardiac Operative Risk Evaluation (EuroSCORE) II), those who had experienced acute MI <2 weeks before surgery, those who had undergone more than one surgical procedure at the same time (including minor procedures with a potential effect on enzyme release, such as left atrial appendage closure and pulmonary vein isolation), and those with severe (preoperative creatinine clearance <30 mL/minute) or end-stage renal disease were excluded.^5^

### Data collection

Data regarding the procedure (on-pump CABG surgery alone) were collected retrospectively from the hospital’s internal data management system, and then the individual dossiers were analyzed after obtaining the relevant ethics approval.

The following data were collected: demographic data (sex, age, body mass, body mass index (BMI)); data regarding comorbidities (obesity (BMI ≥30 kg/m^2^), type I and II diabetes, systemic arterial hypertension, hyperlipidemia, peripheral arterial disease, family history of cardiovascular disease, past or current tobacco use, recent MI (90 to 14 days before surgery), preoperative left ventricular ejection fraction (LVEF; preserved: LVEF ≥50% or altered: mild: 40% to 49%, moderate: 30% to 39%, or severe: <30%), and significant single, double, or triple-vessel coronary disease); surgery-related parameters (direct intrapericardial defibrillation (≥2 shocks of 20 joules), cardiopulmonary bypass, and the duration of aortic cross-clamping); the 30-day postoperative outcomes (all-cause and cardiac-related mortality, stroke, postoperative coronary revascularization (surgical or percutaneous), mechanical hemodynamic support by intra-aortic balloon pump (IABP) or extracorporeal membrane oxygenation (ECMO), sternal re-intervention (because of hemorrhage, infection, or instability), minor complications (atrial fibrillation and nosocomial pneumonia), newly developed renal failure with a glomerular filtration rate ≤50 mL/minute, and the postoperative LVEF status (preserved function, or mild, moderate, or severe dysfunction; and increased or decreased *vs*. the preoperative LVEF), the duration of intubation, and the duration of the intensive care unit (ICU) stay); and the postoperative increases in cardiac enzyme activities (creatinine kinase–myocardial band (CK-MB) and troponin I (cTnI, which was the marker of myocardial ischemia used until July 2013, when it was replaced by high-sensitivity cardiac troponin T (hs-cTnT)); were recorded (for the target group, measurements were made 6, 12, 24, and 48 hours postoperatively, and for the control group 6 and/or 12 hours postoperatively).

The troponin I assay used until 2013 (AccuTnI; Beckman–Coulter, Brea, CA, USA) had the following specifications: the 99^th^ percentile of the upper limit of the reference range (URL) was 0.3 µg/L, the precision (coefficient of variation ≤10%) was 0.06 µg/L, and the detection limit was 0.01 µg/L.^6^ The subsequently used hs-cTnT assay (Elecsys Troponin T high-sensitivity kit; Roche, Basel, Switzerland) had the following specifications: the 99^th^ percentile URL was 14 ng/L, the precision (coefficient of variation ≤10%) was 13 ng/L, the limit of detection was 5 ng/L, and a “blank” containing 3 ng/L was used.^7^ The CK-MB activity (CARDIAC Control CK-MB; Roche) is expressed in international units per liter (U/L), and the assay had a limit of detection of 3 U/L, a “blank” containing 3 U/L was used, and the limit of quantification was 5 U/L.^8^

Pre- and postoperative echocardiography (at hospital admission and 1 week after surgery, respectively) was performed with a focus on new-onset ventricular dysfunction (considered to be significant if it had worsened by at least one grade on the EuroSCORE classification) or regional wall motion abnormalities that were attributable to abnormal coronary perfusion territories.^5^

### Statistical analysis

IBM SPSS v.27.0 for Windows (IBM Corp., Armonk, NY, USA) was used for data analysis. Non-normally distributed datasets are described using the median and interquartile range and normally distributed variables are described using the mean and standard deviation. Owing to the sample size, the use of graphical distribution analyses was preferred. The risk stratifications of the groups were compared using the chi-square (categorical), Student’s *t* (continuous, normally distributed data), or Mann−Whitney U (continuous, non-normally distributed data) tests. The relationships of perioperative MI with the parameters of interest were evaluated using logistic regression. Circulating cardiac enzyme activities were illustrated using scatter plots and compared using boxplots. For the postoperative hs-cTnT and CTnI measurements, a receiver operating characteristic (ROC) analysis, including the calculation of the area under the curve (AUC), was performed to access the predictive ability and discriminatory power of these markers for the correct classification of patients according to their perioperative MI status and to determine the most appropriate threshold values. All the authors had full access to the data and take responsibility for their integrity.

## Results

Of 2825 patients who underwent elective CABG surgery, 78 experienced a perioperative MI, which corresponds to an estimated incidence of 2.8%. After applying the exclusion criteria and further excluding patients who refused consent for the use of their data, 847 patients (71 who experienced perioperative MI and 776 controls) remained for analysis. A family history of cardiovascular disease (37% *vs*. 13% for the target *vs*. the control group, respectively; odds ratio (OR) 2.8; *p* = 0.005), tobacco abuse (54% *vs*. 24%; OR 3.8; *p* < 0.001), recent (90 to 14 days before surgery) MI (27% *vs*. 8%; OR 3.6; *p* < 0.001), and triple-vessel coronary disease (90% *vs*. 74%; OR 2.8; *p* = 0.006) were found to be independent risk factors for MI (Table 1).

**Table 1.** Demographic data, risk factors, perioperative parameters, and outcomes of perioperative MI.

	All patients	Perioperative MI group	Control group				
Number of patients	847	71	776	*P*	Logistic regression		
Demographics/Risk factors							
*Binary variables*	*No. (%)*			*P (χ^2^)*	*OR*	*P*	*95% CI*
Male/Female	706/141 (83.4/16.6)	51/20 (71.8/28.2)	655/121 (84.4/15.6)	0.006		0.338	−0.046–0.135
Obesity (BMI ≥ 30 kg/m^2^)	157 (18.5)	18 (25.4)	139 (17.9)	0.123		0.45	−0.061–0.138
T2DM	151 (17.8)	9 (12.7)	142 (18.3)	0.236		0.182	−0.139–0.027
T1DM	104 (12.3)	8 (11.3)	96 (12.4)	0.786		0.35	−0.116–0.041
High blood pressure	609 (71.9)	52 (73.2)	557 (71.8)	0.793		0.509	−0.046–0.093
Hyperlipidemia	553 (65.3)	52 (73.2)	501 (64.6)	0.142		0.725	−0.054–0.078
Peripheral artery disease	43 (5.1)	5 (7)	38 (4.9)	0.431		0.736	−0.088–0.124
Family history	123 (14.5)	26 (36.6)	97 (12.5)	<0.001	2.809	0.005	0.076–0.237
Smoking history	227 (26.8)	38 (53.5)	189 (24.4)	<0.001	3.78	<0.001	0.059–0.187
Recent MI (90–14 days prior to surgery)	77 (9.1)	19 (26.8)	58 (7.5)	<0.001	3.587	<0.001	0.074–0.255
LVEF ≥50%	543 (64.1)	56 (78.9)	487 (62.8)	0.007		0.811	−0.642–0.503
LVEF 40%–49%	171 (20.2)	7 (9.9)	164 (21.1)	0.022		0.497	−0.78–0.379
LVEF 30%–39%	100 (11.8)	5 (7)	95 (12.2)	0.194		0.355	−0.832–0.299
LVEF <30%	33 (3.9)	3 (4.2)	30 (3.9)	0.881		0.37	−0.86–0.321
Single coronary disease	32 (3.8)	3 (4.2)	29 (3.7)	0.836		0.807	−0.184–0.236
Double coronary disease	181 (21.4)	4 (5.6)	177 (22,8)	<0.001			
Triple coronary disease	634 (74.9)	64 (90.1)	570 (73.5)	0.002	2.775	0.006	0.029–0.172
*Continuous variables*	*Mean ± SD*			*P (t-test)*			
Age (years)	67 ± 10	68 ± 9	67 ± 10	0.276		0.214	−0.006–0.001
Body mass (kg)	80 ± 15	75 ± 15	81 ± 15	0.004		0.055	−0.009–0
BMI (kg/m^2^)	27 ± 5	27 ± 4	27 ± 5	0.168		0.091	−0.002–0.028
Perioperative parameters							
*Binary variables*	*No. (%)*			*P (χ^2^)*			
Defibrillation (≥2 × 20 joules)	41 (4.8)	6 (8.5)	35 (4.5)	0.139		0.83	−0.126–0.101
*Continuous variables*	*Median (IQR)*			*P (MWU)*			
Duration of CPB (min)	86 (68–112)	97 ± 52	90 ± 33	0.029		0.335	−0.001–0.003
Duration of ACC (min)	61 (45–88)	70 ± 38	67 ± 30	0.108		0.461	−0.003–0.001
Postoperative outcomes (30 days)							
*Binary variables*	*No. (%)*			*P (χ^2^)*	*OR*	*P*	*95% CI*
Death	11 (1.3)	3 (4.2)	8 (1)	0.023		0.189	−0.52–0.103
Cardiac death	5 (0.6)	3 (4.2)	2 (0.3)	<0.001	2.341	0.02	0.098–1.128
Stroke	16 (1.9)	3 (4.2)	13 (1.7)	0.131		0.596	−0.251–0.144
IABP	25 (3)	12 (16.9)	13 (1.7)	<0.001	3.967	<0.001	0.153–0.455
ECMO	9 (1.1)	1 (1.4)	8 (1)	0.766		0.991	−0.246–0.243
Sternal re-intervention*	39 (4.6)	6 (8.5)	33 (4.3)	0.106		0.57	−0.302–0.166
Surgical revascularization	12 (1.4)	11 (15.5)	1 (0.1)**	<0.001	4.076	0.001	0.288–0.825
Percutaneous revascularization	8 (0.9)	8 (11.3)	–	<0.001	4.187	<0.001	0.266–0.738
New atrial fibrillation	250 (29.5)	27 (38)	223 (28.7)	0.1		0.66	−0.049–0.077
Pneumonia	74 (8.7)	10 (14.1)	64 (8.2)	0.095		0.544	−0.069–0.129
New renal failure (GFR ≤50 mL/min)	114 (13.5)	17 (23.9)	97 (12.5)	0.007		0.1	−0.189–0.017
LVEF ≥ 50%	531 (63.4)	42 (59.2)	489 (63.8)	0.492		0.159	−1.022–0.168
LVEF 40%–49%	169 (20)	13 (18,3)	156 (20.1)	0.717		0.183	−1.008–0.193
LVEF 30%–39%	113 (13.3)	8 (11.3)	105 (13,5)	0.591		0.179	−0.993–0.186
LVEF <30%	34 (4)	8 (11.3)	26 (3.4)	<0.001		0.688	−0.703–0.464
LVEF increased	22 (2.6)	4 (5.6)	18 (2.3)	0.093		0.153	−0.041–0.263
LVEF decreased	60 (7.1)	19 (26.8)	41 (5.3)	<0.001	4.38	<0.001	0.129–0.339
*Continuous variables*	*Mean ± SD*			*P (t-test)*			
Creatinine clearance (mL/min/1.73 m^2^)	82 ± 36	61 ± 25	85 ± 36	<0.001	−3.441	0.001	−0.004–−0.001
Intubation time (days)	1 (1) [1–32]	2.2 ± 2.3	1.6 ± 1.9	<0.001	3.127	0.002	0.068–0.015
Duration of the intensive care unit stay (days)	1 (1–3) [0–67]	5.6 ± 5	2.4 ± 3.9	<0.001	4.281	<0.001	0.013–0.034

MI, myocardial infarction; No., number; χ^2^, chi-square test; CI, confidence interval; OR, odds ratio; BMI, body mass index; LVEF, left ventricular ejection fraction; SD, standard derivation; IQR, interquartile range; CPB, cardiopulmonary bypass; ACC, aortic cross-clamp; MWU, Mann–Whitney U test; IABP, intra-aortic balloon pump; ECMO, extracorporeal membrane oxygenation; GFR, glomerular filtration rate; T1DM, type 1 diabetes mellitus; T2DM, type 2 diabetes mellitus. * because of hemorrhage, infection, or instability. ** One patient underwent surgical revascularization because of iatrogenic type A dissection, but did not meet the criteria for perioperative MI.

The patients who experienced perioperative MI were characterized by higher cardiac-related mortality (4% *vs*. 0.3% for the target group *vs*. the control group, respectively; OR 2.3; *p* = 0.02), more frequent use of an IABP (17% *vs*. 2%; OR 4; *p* < 0.001), more frequent deterioration of cardiac function on echocardiography (27% *vs*. 5%; OR 4.4; *p* < 0.001), lower creatinine clearance (OR −3.4; *p* = 0.001), longer intubation (OR 3.1; *p* = 0.002), and a longer ICU stay (OR 4.3; *p* < 0.001).

In 26.8% of patients in the perioperative MI group, coronary angiography revealed graft occlusion or stenosis, which was treated surgically (by repeat CABG or anastomotic revision, 15.5%), percutaneously (by stenting of the native vessel, 9.9%), or by anastomotic balloon dilatation (1.4%).

Troponin activity was measured postoperatively in over 86% of the included patients. There were missing data only for the control group, and these were almost exclusively during the initial follow-up period, because troponin measurement was not routinely performed at that time. However, CK-MB activity was measured postoperatively in most of these patients. The median postoperative increase in CTnI was 1.8-fold larger in the MI group than in the control group after 6 hours (2.4 µg/L *vs*. 1.3 µg/L; *p* < 0.001) and 2.7-fold larger after 12 hours (7.8 µg/L *vs*. 2.9 µg/l; *p* = 0.002). The median increases in the hs-cTnT (831 ng/L *vs*. 403 ng/L; *p* < 0.001) and CK-MB (83 U/L *vs*. 46 U/L; *p* < 0.001) activities 12 hours postoperatively were also significantly higher (Supplemental data).

**Figure 1.**
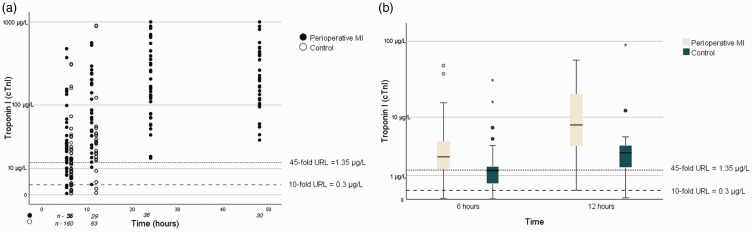
Troponin I activities for each group. (a) Scatter plot of the activities for each group at each time point and (b) box plot of the increases in troponin I activity 6 and 12 hours postoperatively. MI, myocardial infarction; URL, upper limit of the normal range.

**Figure 2.**
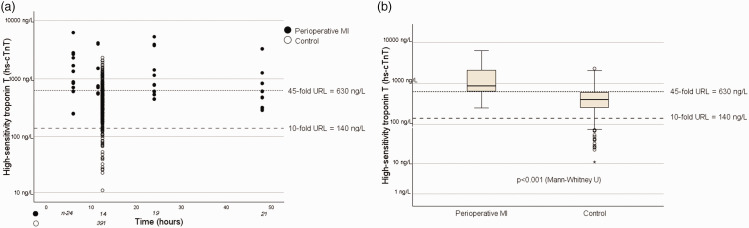
High-sensitivity troponin T activities for each group. (a) Scatter plot of the activities for each group at each time point. (b) Box plot of the postoperative increases in hs-cTNT activity. hs-cTNT, high-sensitivity troponin I activity; MI, myocardial infarction; URL, upper limit of the normal range.

A scatter plot showed that the postoperative cTnI activities of the groups were similarly distributed (Figure 1(a)). Strikingly, 86.4% (at 6 hours) and 88.4% (at 12 hours) of the measurements made for the control group were above the threshold defined for type 5 MI (dashed line, Figure 1(a), (b)). In addition, 91.6% of the hs-cTnT activities of the control group were >10 times the URL (Figure 2(a)), but the boxplot shows a clearer separation of the groups (Figure 2(b)).

The threshold value identified that correctly assigned all the hs-cTnT measurements within the boxes to their respective groups was 45 times the URL (dotted line, Figure 2(b)).

In the MI group, there were no false-negative hs-cTnT values with respect to the type 5 MI threshold (<10 times URL value, according to the ESC guidelines), which confirms the high sensitivity of this marker (Figure 2(a)).

**Figure 3.**
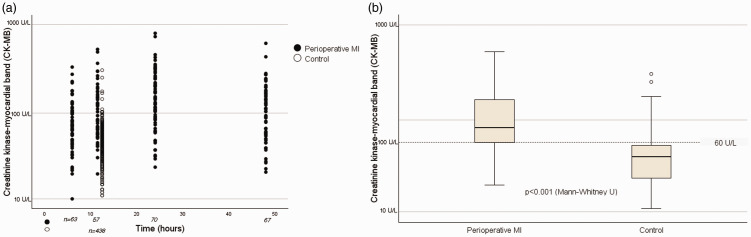
Creatinine kinase–myocardial band activities for each group. (a) Scatter plot of the activities for each group at each time point and (b) box plot of the postoperative increases in CK-MB activity. CK-MB, creatinine kinase–myocardial band; MI, myocardial infarction.

With respect to the postoperative CK-MB activity, the use of a threshold of approximately 60 U/L permitted correct group assignments for 75% of the patients (Figure 3(a), (b)).

**Figure 4.**
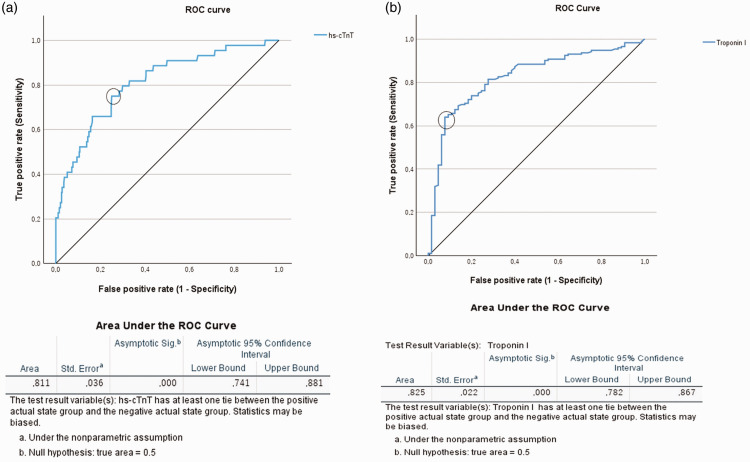
Results of the receiver operating characteristic analysis. ROC curves and areas under the curves for the postoperative increases in (a) troponin I and (b) high-sensitivity troponin T activities. Threshold values at the closest point to the upper left corner of the graph were identified and are circled. hs-cTNT, high-sensitivity troponin I activity; ROC, receiver operating characteristic.

The ROC analysis generated an AUC for hs-cTnT of 0.81, and therefore this parameter met the criterion for a discriminating test. A cut-off value (the closest point to the upper left corner of the graph) of 602 ng/L was identified, which was associated with a sensitivity of 0.75 (75% of all the patients who experienced a perioperative MI were correctly classified) and a 1−specificity of 0.25 (25% of the patients in the control group were incorrectly classified as having experienced a perioperative MI), and this was similar to the 45 × URL value of 620 ng/L that was determined using the boxplot analysis (Figure 4(a)). An AUC of 0.83 was calculated during the ROC analysis for CTnI, confirming that this was also a discriminating means of detecting perioperative MI. A cut-off value of 5.28 µg/L was calculated for this parameter, with a sensitivity of 0.62 and a 1−specificity of 0.08 (Figure 4(b)).

## Discussion

In the present retrospective study, we have shown greater mortality and morbidity in patients undergoing CABG who have experienced a perioperative MI, an association that is well documented.^9^ This means that it is important to have a clear method of classifying patients, based on clinical and biologic parameters, for early diagnosis. Although the criteria for a type 5 MI described in the ESC guidelines (an increase in cardiac troponin of >10 times the URL, ECG changes, and imaging evidence) can help to identify a perioperative MI, they are not specific with regard to its etiology.^1^

In this context, the differentiation of early graft failure (EGF), procedure-related ischemia owing to insufficient myocardial protection, or troponin and CK-MB release owing to direct intraoperative traumatic manipulations, is crucial for the optimal treatment and prognosis of patients.^10^ With respect to the first of these, an immediate intervention to restore coronary flow should be considered because this complication is associated with a high intrahospital mortality rate of up to 22.4%, as reported by Baumgarten *et al*.^11^

To make matters worse, different definitions of perioperative CABG-associated MI have been provided by the various medical societies.^12^ For example, the Society for Cardiovascular Angiography and Intervention (SCAI) considers that the identification of a postoperative CK-MB activity increase of ≥10 times the URL is sufficient for a diagnosis of perioperative MI to be made.^13^

The estimated incidence of perioperative MI was 2.8%, which is consistent with the results of randomized controlled trials of percutaneous *vs*. surgical revascularization.^14,15^ When taken together with a documented incidence of EGF of >25%, the identification of a perioperative MI using the criteria described above seems to be a plausible approach. However, the lack of specificity of the previously defined troponin thresholds for preoperative MI is striking. In contrast, the boxplot analysis showed the correct classification of 75% of the included patients using a 45-fold increase in the hs-cTnT over the URL. This is consistent with the association between the postoperative “abnormal” troponin activities and the specific etiology described by the authors of the ESC Joint Working Groups on Cardiovascular Surgery:

“High cardiac troponin levels … (>45 × URL after 12 hours …), even in the absence of ECG and/or imaging evidence of myocardial infarction, should raise suspicion of early graft failure”.^16^

Finally, the high sensitivity of the marker was confirmed. Thus, if the 10-fold URL threshold is not exceeded, perioperative MI can be reliably excluded, although this threshold was only associated with a correct classification of 8.4% of the patients in the control group.

There have been few studies with a clear focus on the analysis of perioperative MI. For example, the 2018 ESC guideline recommendations with respect to the threshold for an increase in troponin activity that indicates type 5 MI were based on data collected during a prospective analysis comparing the postoperative increases in cTnI and CK-MB in eight patients with perioperative MI with those of 90 patients in a control group.^17^ Together with the results of another prospective, randomized analysis comparing on-pump beating-heart with arrested-heart CABG surgery that included data from 40 patients (eight with new perioperative myocyte necrosis detected by delayed enhancement on postoperative cardiac magnetic resonance imaging and 32 patients with no evidence of periprocedural ischemia), these findings were the rationale for establishing the described thresholds.^18^ However, in addition to the limited number of patients, an assessment of the potential clinical effect of MI, such as a follow-up analysis of postoperative mortality and morbidity rates, was not performed in these studies, and interestingly, the authors of the former study identified a very high threshold for the 12-hour cTnI increase that was indicative of perioperative MI using ROC analysis.^17^ Furthermore, the randomized trial also included patients with ongoing acute coronary syndrome and some who had required emergency interventions, which limits its value in this context.^18^

An important limitation of the present study was that the data were collected retrospectively. The diagnosis of perioperative MI was made after surgery, using the criteria described above, according to the clinical course. Thus, reversible ECG or echocardiographic changes in patients with an otherwise normal postoperative course were generally not classified as having experienced “perioperative infarction,” although “pathologic” myocardial ischemia might have been causative. This may reflect the dilemma of the gradual transition from surgically-induced transient ischemia caused by the factors mentioned above to irreversible myocardial damage, which makes a clear classification difficult to achieve. Moreover, coronary angiography was, of course, not performed in the patients in the control group, but neither was it performed in all of the patients who experienced perioperative MI, and the criteria for angiographic control were either not established or changed during the long observation period. The treatment of EGF has also changed considerably during the 20 years of the study, with a percutaneous approach now being in general preferred to surgical revision, which may have affected the morbidity and mortality rates. Therefore, further prospective studies should be performed that include the systematic imaging of all patients who undergo CABG surgery alone, to better characterize the severity and cause of perioperative MIs. As in the randomized study by Pegg *et al*., this could be achieved using pre- and postoperative gadolinium-enhanced cardiac magnetic resonance imaging, and in the case of newly occurring ischemia, by supplementary coronary computed tomography to reliably distinguish perioperative MI from the other possible causes of periprocedural ischemia mentioned above and to accurately determine the incidence of EGF.^18^

## Conclusions

Based on the available data, we can draw the following conclusions. First, the diagnostic value of the postoperative increases in cardiac enzyme activity alone for the reliable identification of perioperative MI appears to be limited. Second, the consideration of imaging and ECG changes in addition to the postoperative enzyme increases seems to permit more accurate identification of perioperative MI, which in the present study was associated with higher mortality, morbidity, and graft occlusion rates. Third, an increase of 45 times the URL for hs-cTnT 12 hours following the procedure may indicate a perioperative MI and should prompt further imaging to rule out EGF if there are concomitant ECG and/or echocardiographic changes. Finally, a postoperative hs-cTnT increase of <10 times the URL is probably sufficient to rule out perioperative MI, although this classification is only likely to be useful in a modest proportion of patients who show a normal postoperative course.

## Supplemental Material

sj-pdf-1-imr-10.1177_03000605241306866 - Supplemental material for Perioperative infarction during coronary bypass surgery: an attempt to refine the diagnostic criteria using data from a retrospective case–control study Supplemental material, sj-pdf-1-imr-10.1177_03000605241306866 for Perioperative infarction during coronary bypass surgery: an attempt to refine the diagnostic criteria using data from a retrospective case–control study by Lars Niclauss, Liam-Kani Roulet, Piergiorgio Tozzi, Filip Dulguerov, Ziyad Gunga, Anna Nowacka and Matthias Kirsch in Journal of International Medical Research
