# Supplementary material for: Perioperative infarction during coronary bypass surgery: an attempt to refine the diagnostic criteria using data from a retrospective case–control study
Source: J Int Med Res. 2025 Jan 27;53(1):03000605241306866. doi: 10.1177/03000605241306866 (PMC11770777; doi:10.1177/03000605241306866)
Supplement: sj-pdf-1-imr-10.1177_03000605241306866 - Supplemental material for Perioperative infarction during coronary bypass surgery: an attempt to refine the diagnostic criteria using data from a retrospective case–control study [file sj-pdf-1-imr-10.1177_03000605241306866.pdf]

Group		All	Perioperative MI	Control	
**Number of patients**		**847**	**71**	**776**	**P**
**Heart enzymes**					
Continuous			*Median (IQR)*		*(M-Whitney U)*
cTnI (µg/l)	6 h	1.6 (0.8 - 3)	2.4 (1.4 - 5)	1.3 (0.6 -1.6)	< 0.001
	12 h	4 (2 - 12.9)	7.8 (3.3 - 20.6)	2.9 (1.5 - 4)	0.002
Hs-cTnT (ng/l)	12 h	434 (269 – 651)	831 (571 - 1601)	403 (259 - 603)	< 0.001
CK-MB (U/l)	12 h	48 (37 - 63)	83 (58 - 170)	46 (36 - 57)	< 0.001
CK (U/l)	12 h	295 (217- 427)	629 (390 – 1242)	268 (208 – 366)	< 0.001

MI - myocardial infarction; IQR - interquartile range; cTnI - troponin I; hs-cTnT - high-sensitive cardiac troponin T; CK-MB - creatinine kinase-myocardial band; CK - creatinine kinase
